# Capsaicin suppresses LPS-induced inflammatory responses via NLRP3/CASP-1/IL-1β axis and purinergic pathways in BV-2 microglial cells

**DOI:** 10.1007/s11302-025-10123-5

**Published:** 2026-01-12

**Authors:** Bianca Vedoin Copês Rambo, Milagros Fanny Vera Castro, Mairin Schott, Robson Lourenço da Silva Santos, Charles Elias Assmann, Marcylene Vieira da Silveira, Pâmela de Almeida Milioni, Adriel Antonio Schirmann, Vitor Bastianello Mostardeiro, Nathieli Bianchin Bottari, Maria Rosa Chitolina Schetinger, Vera Maria Melchiors Morsch

**Affiliations:** 1 Postgraduate Program in Biological Sciences: Toxicological Biochemistry, Department of Biochemistry and Molecular Biology, Federal University of Santa Maria (UFSM), Santa Maria, RS Brazil; 2 Postgraduate Program in Microbiology and Parasitology, Department of Microbiology and Parasitology, Federal University of Pelotas (UFPEL), Pelotas, RS Brazil

**Keywords:** Capsaicin, Microglia, Neuroinflammation, Lipopolysaccharide, Purinergic System

## Abstract

Microglial activation drives neuroinflammation, a key factor in many neurological diseases. The purinergic system is a major regulator of inflammatory responses and represents a promising target for controlling neuroinflammation. Capsaicin, a bioactive compound found in chili peppers, exhibits significant anti-inflammatory and antioxidant properties. This study aimed to investigate the modulatory effects of capsaicin on microglial activation and purinergic system regulation. For this, BV-2 microglial cells were exposed to lipopolysaccharide (1 μg/mL) and treated with capsaicin (25 and 50 μM) for 24 hours. Cell viability was assessed by MTT and trypan blue assays. Cell cycle and apoptosis were evaluated by flow cytometry. Nitric oxide, reactive species and malondialdehyde levels were evaluated as markers of oxidative stress. Activities of NTPDase, 5'-nucleotidase (5’-NT), and adenosine deaminase (ADA) were evaluated. Gene expression of inflammatory mediators and purinergic receptors were analyzed by qRT-PCR, and molecular docking analyses were performed. As a result, capsaicin decreased the expression of pro-inflammatory mediators (NLRP3, CASP-1, IL-1β, IL-6, and TNF-α), increased IL-10 expression, and attenuated oxidative stress. It reduced NTPDase, 5'-NT, and ADA activities, downregulated P2X7 and A2A receptor expression, and upregulated A1 receptor expression. Molecular docking revealed that capsaicin has a high affinity for the A1 and A2A receptors, as well as for ADA. Collectively, these findings suggest that capsaicin exerts neuroprotective effect by suppressing pro-inflammatory signaling, enhancing anti-inflammatory responses, reducing oxidative stress, and modulating key components of the purinergic system, including ectoenzyme activities and P2X7, A1, and A2A receptor expression.

**Graphical Abstract:**

Lipopolysaccharide (LPS) activates microglia via the NLRP3 pathway, increasing pro-inflammatory cytokines, purinergic receptors expression (P2X7 and A2A), and the activity of CD39, CD73, and ADA. Capsaicin mitigates this inflammatory profile by inhibiting NLRP3 signaling, reducing pro-inflammatory cytokine expression and modulating purinergic signaling toward an anti-inflammatory profile. Illustrations were obtained from Servier Medical Art (https://smart.servier.com/), licensed under the Creative Commons Attribution 4.0 International License (CC BY 4.0).

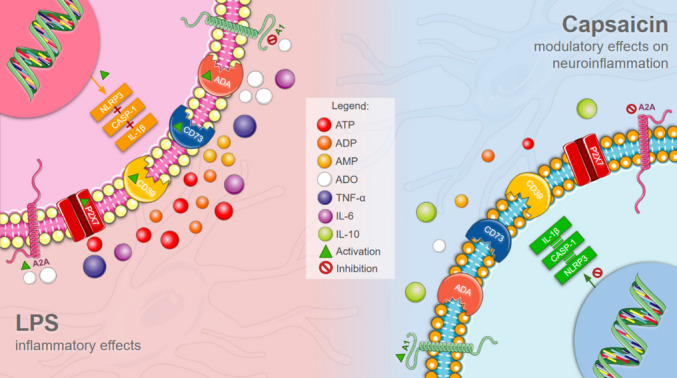

## Introduction

Neuroinflammation is a response involving reactive components within the central nervous system (CNS) due to disruptions in homeostasis caused by endogenous or exogenous stimuli. This response is characterized by the release of inflammatory mediators that contribute to restoring tissue structure and function [1]. Many neurological disorders, such as traumatic brain injury, neoplasms, ischemia, metabolic diseases, autoimmune diseases, infections of the nervous system, and neurodegenerative diseases, are associated with neuroinflammatory processes [2, 3].

In response to harmful stimuli and cellular changes, microglia become activated and release inflammatory mediators, including cytokines and chemokines. These substances are involved in the recruitment of additional cells and the elimination of pathological agents [4, 5]. Simultaneously, microglia promote tissue repair and regeneration by releasing anti-inflammatory cytokines and growth factors. Consequently, the regulated activation of microglia during the inflammatory response has been demonstrated to yield beneficial outcomes, aiding in the maintenance of homeostasis within the nervous system [6, 7].

However, sustained inflammatory responses and chronic microglial activation are detrimental, ultimately leading to neurotoxicity. Inflammatory stimulation can persist due to a combination of endogenous (e.g., genetic predisposition and protein aggregation) and environmental (e.g., infection, trauma, or drugs) factors [8, 9]. In this state, microglia remain activated in a pro-inflammatory phenotype, resulting in increased activation of inflammatory signaling pathways and excessive production of neurotoxic and pro-inflammatory mediators, such as nitric oxide (NO), IL-6, IL-1β, tumor necrosis factor alpha (TNF-α), and reactive species (RS). Consequently, these factors contribute to demyelination and, ultimately, neurodegeneration [3–5].

The purinergic system is one of the mechanisms responsible for regulating inflammation. It uses nucleotides and nucleosides, such as adenosine triphosphate (ATP) and its metabolites – adenosine diphosphate (ADP), adenosine monophosphate (AMP), and adenosine – to mediate cellular communication in the CNS [10]. Extracellular ATP, released under conditions of cellular stress, tissue damage, or inflammation, acts as a danger signal by activating microglial purinergic receptors [11]. Among these receptors, P2X7 (P2X7R) is particularly notable for its central role in amplifying the inflammatory response. This ATP-sensitive ion channel is highly expressed in activated microglial cells. When stimulated by sustained high concentrations of ATP, it induces the formation of a high-conductance pore [12, 13]. This mechanism promotes an influx of calcium (Ca^2+^) and efflux of potassium (K^+^), leading to a reduction in intracellular K^+^ levels. This reduction subsequently activates the NLRP3 inflammasome, resulting in enhanced release of pro-inflammatory cytokines [13–15].

Concomitantly, extracellular ATP can be hydrolyzed into adenosine through the action of ectoenzymes such as NTPDase (CD39), which catalyzes the hydrolysis of ATP to ADP and ADP to AMP, and 5'-nucleotidase (5'-NT/CD73), which converts AMP into adenosine [11]. Adenosine exerts a modulatory effect by binding to A1 receptors (A1R) and A2A receptors (A2AR). These receptors modulate microglial activation in distinct ways. Activation of A1R has been associated to neuroprotective effects, while stimulation of A2AR has been shown to promote pro-inflammatory responses [16, 17]. Therefore, purinergic receptors play a fundamental role in neuroinflammation and microglial activation, and maintaining a balanced state within this system is essential for preserving brain homeostasis [18].

The search for natural compounds with neuroprotective properties has emerged as a promising strategy for modulating neuroinflammation. Capsaicin (8-Methyl-N-vanillyl-*trans*−6-nonenamide), a vanilloid found in chili peppers of the *Capsicum* genus, has attracted considerable attention for its anti-inflammatory and antioxidant properties [19, 20]. As a Transient Receptor Potential Vanilloid 1 (TRPV1) channel agonist, capsaicin modulates nociceptive sensory responses and signaling pathways associated with neuroinflammation. Recent studies indicate that capsaicin offers therapeutic benefits for neurological and neurodegenerative diseases, with effects involving both TRPV1-dependent and independent pathways [21–25].

Despite extensive research on capsaicin's benefits, its effects on the purinergic system remain inadequately understood. Therefore, this study aimed to investigate the effects of capsaicin on microglial activation in an in vitro model of neuroinflammation induced by lipopolysaccharide (LPS) and its impact on the regulation of the purinergic system.

## Materials and methods

### Reagents and equipment

Capsaicin (≥ 95% pure, CAS 404–86-4, Cat. No. M2028) was purchased from Sigma-Aldrich (St. Louis, MO, USA). All other chemicals and reagents used in the study were of analytical grade and high purity, obtained from Sigma-Aldrich Inc. (St. Louis, MO, USA), Merck KGaA (Darmstadt, Germany), Pharmingen™ BD Biosciences (San Diego, CA, USA), Invitrogen Life Technologies (Carlsbad, CA, USA) Thermo Fisher Scientific (New York, NY, USA), Santa Cruz Biotechnology (Dallas, TX, USA), Bio-Rad Laboratories (Hercules, CA, USA), and Labsynth Produtos para Laboratórios Ltda (Diadema, SP, Brazil).

Cell culture materials were obtained from Corning Inc. (Corning, NY, USA), Gibco® (Thermo Fisher Scientific, Grand Island, NY, USA), Vitrocell Embriolife (Campinas, SP, Brazil), and Kasvi (São José do Pinhais, PR, Brazil).

Spectrophotometric analyses were performed using the SpectraMax® i3 Multimode Plate Reader (Molecular Devices, Sunnyvale, CA, USA). Flow cytometric analyses were conducted on a BD FACsVerse™ flow cytometer (BD Biosciences, San Diego, CA, USA), and quantitative reverse transcription polymerase chain reaction (qRT-PCR) analyses were performed on a QuantStudio 3 equipment (Applied Biosystems, Thermo Fisher Scientific, Waltham, MA, USA).

### Cell culture and treatment protocols

#### Cell culture

BV-2 microglial cells (code 0356), derived from mouse brain tissue, were obtained from the Rio de Janeiro Cell Bank (BCRJ, Rio de Janeiro, Brazil). The cells were cultured in Roswell Park Memorial Institute medium (RPMI-1640) containing 2 mM L-glutamine, 4500 mg/L glucose, 1 mM sodium pyruvate, 1500 mg/L sodium bicarbonate, 10% fetal bovine serum (FBS), and 1% penicillin–streptomycin (10.000 U/mL; 10 mg/mL). Cultures were maintained under standard conditions (37 °C with 95% oxygen and 5% CO_2_). For analysis, cells were seeded at a density of 3 × 10^5^ cells/mL.

#### Concentration–response curve of capsaicin

To evaluate the concentration–response of capsaicin, cells were treated with various concentrations (1, 5, 10, 25, 50, 75, 100, 200 and 300 μM) for 24 and 48 hours. Due to the hydrophobic nature of capsaicin, it was solubilized in the culture medium using 0.4% dimethyl sulfoxide (DMSO) [26–28]. The control group received culture medium only, and the DMSO group received 0.4% DMSO to assess solubilizer toxicity.

#### Treatment

Capsaicin concentrations of 25 and 50 μM were selected based on the analysis of the concentration–response curve. These concentrations were subsequently used in the experimental procedures. To mimic neuroinflammation, cells were exposed to lipopolysaccharide (LPS; *Escherichia coli* O111:B4, Sigma-Aldrich, Cat. No. L4391) at a concentration of 1 μg/mL [29, 30]. Cells were simultaneously exposed to LPS and capsaicin (25 and 50 μM) and incubated for 24 hours. The experimental groups and treatments are presented in Table 1. All experiments were performed in triplicate in three independent trials (*n* = 3).

**Table 1:** Experimental groups and treatments

Group	Treatment
Ctrl	Control, untreated cells
CAP 25 μM	Capsaicin 25 μM
CAP 50 μM	Capsaicin 50 μM
LPS	LPS 1 μg/mL
LPS + CAP 25 μM	LPS 1 μg/mL + Capsaicin 25 μM
LPS + CAP 50 μM	LPS 1 μg/mL + Capsaicin 50 μM

### Cell viability assays

Cell viability was assessed using the MTT and Trypan Blue assays. The MTT (3-(4,5-dimethyl-2-thiazolyl)−2,5-diphenyl-2H-tetrazolium bromide; Sigma-Aldrich) assay was performed following a previously described method [31]. BV-2 cells were seeded in 96-well plate and treated according to the experimental design. After treatment, cells were incubated with 5 mg/mL MTT for 4 h at 37 °C. DMSO was then added to solubilize the formazan crystals, and the absorbances were measured at 570 nm. Results were expressed as the percentage relative to the control group.

The Trypan Blue exclusion assay was performed in accordance with the published guidelines, with adaptations [32]. BV-2 cells were seeded in 48-well plate. After treatments, cells were detached using Trypsin–EDTA 1X. To inhibit trypsin activity, an equal volume of culture medium containing 10% FBS was immediately added after detachment. The cell suspension was then mixed 1:1 with 0.4% Trypan Blue stain (Gibco). Subsequently, 10 μL of the mixture was transferred to the CYTO C-Chip chamber (inCYTO C-Chip, DHC-N01-5, Neubauer Improved), and live and dead cells were counted using a bright-field microscope at 20X magnification. Results were expressed as the percentage relative to the control group.

The half-maximal inhibitory concentration (IC_50_) of capsaicin in BV-2 cells was assessed using MTT assay data. IC_50_ values were calculated using GraphPad Prism 9 software (equation: log(inhibitor) vs normalized response – variable slope). IC_50_ values are reported along with 95% confidence intervals (CI 95%).

### Flow cytometry analysis

#### Cell cycle

Cell cycle phases were evaluated following a previously published method [31]. BV-2 cells were seeded in a 6-well plate at a final concentration of 1 × 10^5^ cells/mL and treated according to the experimental design. After the incubation period, cells were harvested using a trypsin solution. Culture medium containing 10% FBS was added to inhibit trypsin activity. Subsequently, the samples were washed with 1X Phosphate Buffered Saline (PBS), and the cell pellet was resuspended and fixed in 70% ethanol and incubated at −20 °C overnight. Prior to analysis, samples were centrifuged, washed with 1X PBS and stained with 500 μL of propidium iodide (PI; BD Biosciences) solution (300 μL of 1 × PBS containing 0.05% Triton X-100, 100 μL of RNase at 100 mg/mL, and 100 μL of PI). The cells were incubated at 37 °C for 40 min, then washed and resuspended in 500 μL of 1X PBS for subsequent flow cytometry analysis. The percentages of cells in the G0/G1, S, and G2/M phases were analyzed using FlowJo vX 10.6 software (Tree Star Inc., Ashland, OR, USA).

#### Apoptosis

To assess apoptosis, a FITC-Anexin-V and propidium iodide (PI) staining kit (BD Biosciences) was used [33]. FITC-Annexin V identifies apoptotic cells, while PI distinguishes dead cells. Viable cells are negative for both FITC-Annexin V and PI, early apoptotic cells are FITC-Annexin V positive and PI negative, and late apoptotic or necrotic cells are positive for both markers. The assay was performed according to the manufacturer’s instructions. Briefly, BV-2 cells were seeded in 6-well plates at a density of 1 × 10^5^ cells/mL. After 24 h of treatment, cells were detached using a trypsin solution. Culture medium containing 10% FBS was added to inhibit trypsin activity. Subsequently, the samples were washed twice with cold PBS and resuspended in 1X Binding Buffer. For staining, 100 μL of the cell suspension was incubated with 5 μL FITC-Annexin V and 5 μL PI. Cells were gently vortexed and incubated in the dark at room temperature (25 °C) for 15 min. After incubation, samples were centrifuged, resuspended in 500 μL PBS, and immediately analyzed by flow cytometry. The results were analyzed using FlowJo vX 10.6 software (Tree Stars Inc.) and presented as the percentage of apoptotic cells relative to the total cell population.

### Oxidative parameters

#### Nitric oxide

Nitric oxide (NO) production was evaluated by measuring nitrite levels in the supernatant using a colorimetric assay with Griess reagent [34]. In a 96-well plate, 100 μL of cell culture supernatant and 100 μL of Griess reagent (0.1% naphthylethylenediamine and 1% sulfanilamide in 5% phosphoric acid (H_3_PO_4_)) were added. After 15 min of incubation in the dark, the absorbances were measured at 540 nm. Results were expressed as the percentage relative to the control group.

#### Reactive species

Intracellular reactive species (RS) levels were quantified using 2′,7′-dichlorofluorescein diacetate (DCFH-DA, 1.87 mM; Sigma-Aldrich), following a previously described method [35]. BV-2 cells were seeded in 6-well plate and treated according to the experimental design. After treatment, 50 μL of the cell suspension were transferred to black 96-well plates. Subsequently, 230 μL of 10 mM Tris–HCl buffer (pH 7.4) and 20 μL of the DCFH-DA solution (diluted in absolute ethanol) were added. The fluorescence intensity of DCF was recorded at 488 nm excitation and 525 nm emission immediately after DCFH-DA addition, with kinetic reading performed for 50 min at 30-s intervals. The results were expressed as U DCFH per mg of protein. The total protein concentration was standardized within a range of 0.6–1.0 mg/mL.

#### Lipid peroxidation

The level of thiobarbituric acid reactive substances (TBARS) was performed according to a previously described method [36]. This assay quantifies malondialdehyde (MDA), a marker of lipid peroxidation, through its reaction with thiobarbituric acid (TBA). Briefly, glass test tubes were prepared by adding the following reagents in sequence: 50 μL distilled water, 100 μL sample, 250 μL acetate buffer, 100 μL SDS (8.1%), and 250 μL TBA (0.8%). An MDA (0.3 mM) standard curve was prepared using increasing MDA concentrations (0–300 μL) following the same sequence of reagents. All tubes were incubated in a water bath at 95 °C for 90 min. After incubation, 200 μL of each sample was transferred to a 96-well microplate, and absorbance was measured at 532 nm. Results were expressed as nmol MDA per mg of protein, with the total protein concentration standardized at 1.0 mg/mL.

### Activities of the enzymes of the purinergic system

#### NTPDase and 5′-nucleotidase activity

The activity of the enzymes NTPDase [37] and 5’-NT [38] was evaluated by measuring the release of inorganic phosphate (Pi). Briefly, the cells were centrifuged for 5 min at 200 × g and dissociated into a single cell suspension with saline solution (NaCl, 0.9%). Subsequently, 20 μL of sample was added to the reaction system for each enzyme and pre-incubated for 10 min at 37 °C. The reactions were initiated by adding the specific substrates for each enzyme (ATP and ADP for NTPDase and AMP for 5'-NT). The reactions were terminated by the addition of trichloroacetic acid (TCA, 10%). The Pi released from the hydrolysis of ATP, ADP, and AMP was subsequently quantified using the malachite green colorimetric assay. A standard curve was prepared using Monopotassium Phosphate (KH_2_PO_4_). Absorbance was measured at 630 nm, and enzyme activities were expressed as nmol Pi released per minute per mg of protein. Total protein concentration was standardized within the range of 0.6–0.8 mg/mL.

#### Adenosine deaminase activity

The activity of adenosine deaminase (ADA) was measured by detecting the ammonia produced when the enzyme acted on excess adenosine [39]. For the assay, 50 μL of cell suspension was incubate with 21 mmol/L adenosine (pH 6.5) for 60 min at 37 °C. The reaction was stopped by adding a solution containing 106.2 mM phenol, 167.8 mM sodium nitroprusside, and sodium hypochlorite. Ammonia production was measured at 620 nm, and results were expressed as units of adenosine per mg of protein (U Ado/mg protein). Total protein concentration was standardized within the range of 0.6–0.8 mg/mL.

### Gene expression

Gene expression analysis of cytokines (IL-1β, IL-6, IL-10, TNF-α), NLRP3, Caspase-1 (CASP-1), and the purinergic receptors P2X7, A1, and A2A was performed using qRT-PCR [40]. RNA was isolated using TRIzol® reagent (Invitrogen™) and quantified at 260 nm using a NanoDrop™ 1000 spectrophotometer (Thermo Fisher Scientific). Reverse transcription was performed using the iScript cDNA synthesis kit (Bio-Rad Laboratories) and the RNA added to a final concentration of 1 μg/μL. The following steps were used for cDNA synthesis: 37 °C for 5 min, heating to 65 °C for 10 min and cooling for 10 min at 5 °C. This was followed by an incubation step at 25 °C for 5 min, 42 °C for 30 min, 85 °C for 5 min and a final incubation at 5 °C for 60 min.

qRT-PCR reactions were performed using 9.5 μL of a mix containing iTaq Universal SYBR Green Supermix (Bio-Rad Laboratories) and 0.5 μL of cDNA. The reaction occurred under the following conditions: 95 °C for 3 min, followed by 40 cycles at 95 °C for 10 s, 60 °C for 30 s followed by a melting curve from 65 °C to 95 °C. The beta actin gene (ACTB) was used as a normalizer. The relative expression of each gene was represented as the fold expression compared to the control group and calculated using the comparative ^*ΔΔ*^ CT value. Forward and reverse primer sequences for each gene analyzed are listed in Table 2.

**Table 2:** Forward and reverse primer sequences

Gene	Gene ID	Direction	Primer sequence (5’−3’)	Amplicon size
ACTB	11461	Forward	CCGTAAAGACCTCTATGCCAAC	100 bp
		Reverse	AGGAGCCAGAGCAGTAATCT	
NLRP3	216799	Forward	CCCATACCTTCAGTCTTGTCTTC	84 bp
		Reverse	CTGCCACAAACCTTCCATCTA	
CASP-1	12362	Forward	CTGAGGGCAAAGAGGAAGCA	534 bp
		Reverse	AACTTGAGCTCCAACCCTCG	
IL-1β	16176	Forward	GGTACATCAGCACCTCACAA	124 bp
		Reverse	TTAGAAACAGTCCAGCCCATAC	
IL-6	16193	Forward	CTTCCATCCAGTTGCCTTCT	134 bp
		Reverse	CTCCGACTTGTGAAGTGGTATAG	
IL-10	16153	Forward	ACAGCCGGGAAGACAATAAC	116 bp
		Reverse	CAGCTGGTCCTTTGTTTGAAAG	
TNF-α	21926	Forward	TTGCTCTGTGAAGGGAATGG	95 bp
		Reverse	GCTCTGAGGAGTAGACAATAAAG	
P2X7R	18439	Forward	AAGGCATAGCAGAGGTGACG	110 bp
		Reverse	GAATGAGTTCCCCTGCAAAGG	
A1R	11539	Forward	ACTTCTTCGTCTGGGTGCTG	523 bp
		Reverse	TGAGGAGGAACAGTGGGACA	
A2AR	11540	Forward	ATGGCTTGGTGACGGGTATG	502 bp
		Reverse	ACGGAATTGCTGTGGGAGAG	

### Protein determination

The protein content of the samples was measured using the Coomassie Blue reagent, following a previously described method [41], with bovine serum albumin as the standard. Protein concentrations (mg/mL) were adjusted according to the specific requirements of each assay.

### Homology modeling of *Mus musculus* enzymes and purinergic receptors and acquisition of the three-dimensional structure of capsaicin

The crystallographic structure of capsaicin (C_18_H_27_NO_3_) was obtained from the Cambridge Structural Database (CSD) under the identifier FABVAF01 and deposition number 171602 and was used for the simulations. The crystallographic structure of the enzyme adenosine deaminase (ADA, EC 3.5.4.4) code 1a4m deposited in the PDB was used for the simulations. The three-dimensional structures of the enzymes and purinergic receptors NTPDase (CD39, EC 3.6.1.5), 5'-nucleotidase (CD73, EC 3.1.3.5), A1R, A2AR and P2X7R were obtained by homology modeling from the FASTA sequences of codes AAH11278.1, AAC13542.1, NP_001269874.1, NP_001318024.1 and NP_035157.2, respectively, made available in GenBank. The models were obtained using the online software Swiss-Model, assuming as templates the crystals 3zx2, 6tve, 5uen, 8gne and 8tr6, deposited in the Protein Data Bank (PDB). The models were validated using the MolProbity Score and the Ramachandran map generated by Swiss-Model.

### Molecular docking analysis

The models obtained by homology modeling for CD39, CD73, A1R, A2AR and P2X7R and the 1a4m model of the adenosine enzyme available in the PDB were previously prepared by removing interferents such as ligands and water molecules using the Biovia Discovery Studio v.24.1.0.23298 software. Subsequently, the protonation state of the protein receptors was adjusted to physiological pH (7.4) on the online H + + server and the Kollman charges were added using the Autodock Tools v.1.e5.6 software. Blind docking was then simulated using AutoDock Vina v.1.1.2 where the ligand was considered semi-flexible and the receptors were considered rigid with a grid box of 1 Å for all receptors with dimensions that allowed the entire structure of the receptors to be inside the box and an exhaustiveness equal to 64 was considered for the simulations in AutoDock Vina.

### Statistical analysis

Statistical analyses were performed using GraphPad Prism 9.0 software (GraphPad, La Jolla, CA, USA). Data are presented as the mean ± standard error of the mean using one-way or two-way analysis of variance (ANOVA) followed by Tukey's multiple comparisons test. Statistically significant differences were considered when *P* < 0.05.

## Results

### Concentration–response curve of capsaicin in BV-2 cells

**Fig. 1:**
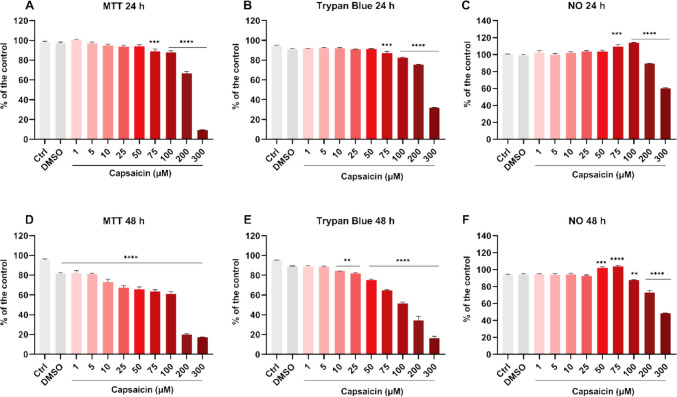
In vitro effects of different concentrations of capsaicin (1–300 μM) on cell viability and nitric oxide levels in BV-2 microglial cells after 24 and 48 hours of incubation. Cell viability assessed by MTT and Trypan Blue assay. Nitric oxide (NO) levels in supernatant. Ctrl = negative control; DMSO = vehicle control (dimethyl sulfoxide, 0.4%). Values expressed as percentage of the negative control ± standard error. One-way ANOVA followed by Tukey's multiple comparisons test. ** P < 0.01, *** P < 0.001 and **** P < 0.0001

The concentration–response curve of capsaicin was assessed by cell viability and nitric oxide levels. After 24 hours of capsaicin treatment, no significant changes in cell viability were observed at 1–50 μM concentrations using the MTT assay. However, viability decreased at 75 μM (*P* = 0.0006), 100 μM (*P* < 0.0001), 200 μM (*P* < 0.0001), and 300 μM (*P* < 0.0001) (Fig. 1A), which was also confirmed by the Trypan Blue assay (Fig. 1B). The IC_50_ value was 219.5 μM (Table 3). Nitric oxide levels remained unchanged at 1–50 μM but increased at 75 μM (*P* = 0.0002) and 100 μM (*P* < 0.0001), then decreased at 200 μM (*P* < 0.0001) and 300 μM (*P* < 0.0001) (Fig. 1C).

**Table 3:** IC_50_ values and 95% confidence intervals (CI)

Time	IC_50_	CI 95% (IC_50_)	LogIC_50_	CI 95% (LogIC_50_)
24 h	219.5	212.1 to 226.7	2.341	2.327 to 2.355
48 h	85.32	66.69 to 113.5	1.931	1.824 to 2.055

Following a 48-hours treatment period, a decrease in cell viability, as assessed by the MTT assay, was observed in all experimental groups (*P* < 0.0001) (Fig. 1D). Trypan Blue assay confirmed a reduction in viable cells starting at 10 μM (10 μM, *P* = 0.0079; 25 μM, *P* = 0.0014; 50–300 μM, *P* < 0.0001) (Fig. 1E). The IC_50_ value was 85.32 μM (Table 3). Nitric oxide levels demonstrated alterations from the concentration of 50 μM (50 μM, *P* = 0.0002; 75 μM, *P* < 0.0001; 100 μM, *P* = 0.0041; 200 and 300 μM, *P* < 0.0001) (Fig. 1F).

Based on these preliminary data, the maximum doses that did not alter cell viability, i.e., 25 μM and 50 μM at 24 hours, were selected for subsequent experiments.

### Capsaicin and LPS do not affect the viability of BV-2 cells

**Fig. 2:**
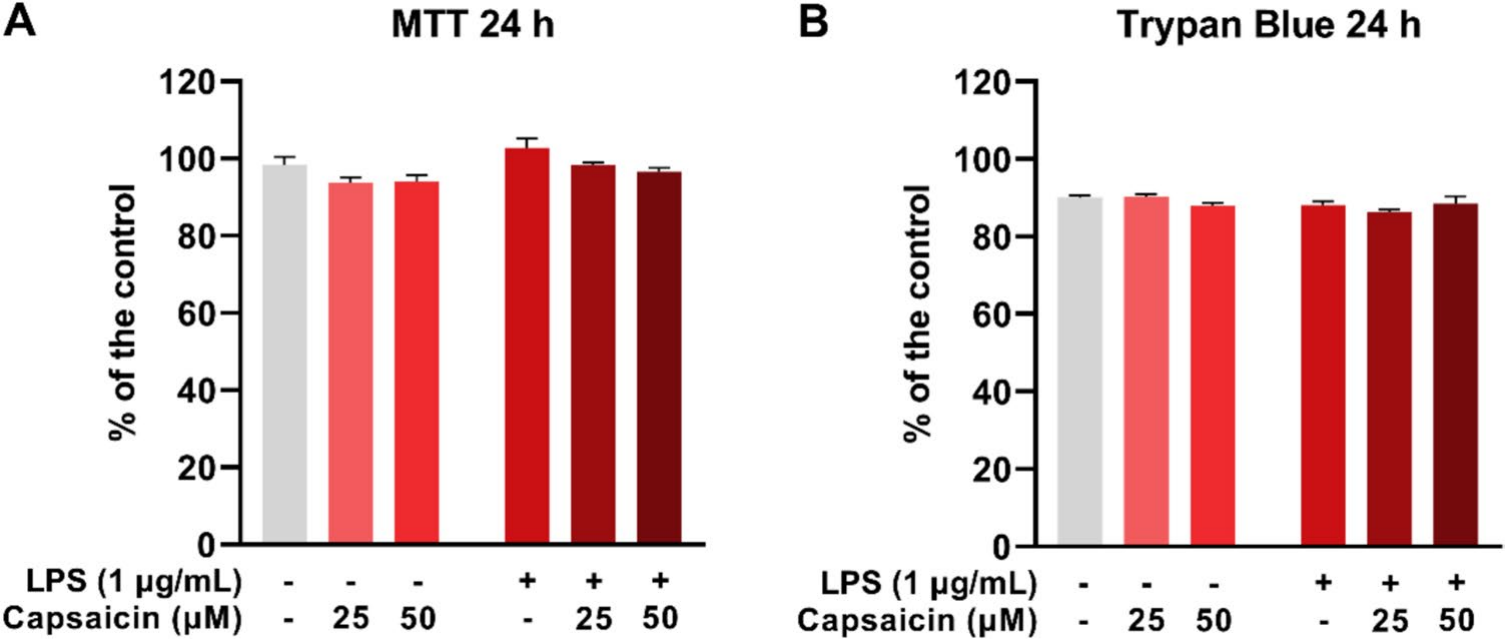
In vitro effects of capsaicin (25 and 50 μM) and lipopolysaccharide (LPS, 1 μg/mL) on cell viability of microglial cells (BV-2) after 24 hours of incubation. Cell viability assessed by MTT (**A**) and Trypan Blue assay (**B**). Values expressed as percentage of the negative control ± standard error. Statistical significance when *P*< 0.05 (two-way ANOVA followed by Tukey's multiple comparisons test)

BV-2 cells were exposed to LPS (1 μg/mL) to induce inflammation and treated with capsaicin (CAP) at concentrations of 25 and 50 μM for 24 hours. Analysis of cell viability revealed no statistically significant differences between the treated groups and the control group (Fig. 2A and B).

**Fig. 3:**
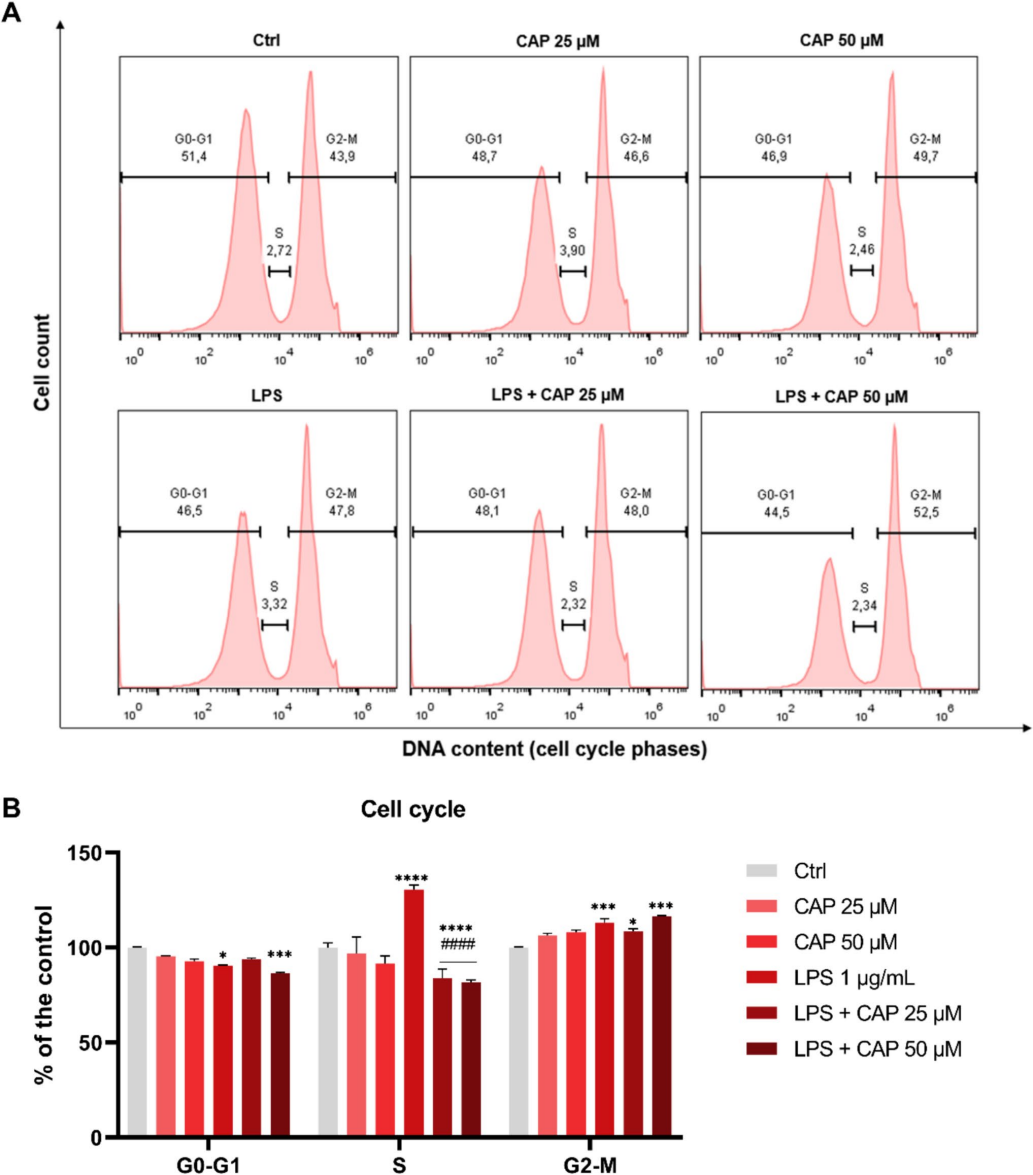
Cell cycle profile of microglial cells (BV-2) treated with or without LPS and capsaicin (25 μM and 50 μM) for 24 hours. Representative DNA histogram of control and treatment groups (**A**). DNA content in each phase of the cell cycle relative to the control group (*n* = 3) (**B**). Ctrl = negative control; LPS = lipopolysaccharide; CAP = capsaicin. Values are expressed as mean ± standard error. Statistical significance when *P* < 0.05 (two-way ANOVA followed by Tukey's multiple comparisons test). *Compared to the control group (*P < 0.05, ***P < 0.001 and ****P < 0.0001). # Compared to LPS group (####*P* < 0.0001)

In the cell cycle analysis (Fig. 3B), the percentage of BV-2 cells in the G0-G1 phase was reduced in the groups treated with LPS (*P* = 0.0168) and LPS + CAP 50 μM (*P* = 0.0007) compared to the control. A significant increase in the S phase was observed in the LPS group (*P* < 0.0001) compared to the control. In contrast, the groups treated with LPS + CAP 25 μM (*P* < 0.0001) and LPS + CAP 50 μM (*P* < 0.0001) exhibited a reduction in this phase compared to the LPS group. In the G2-M phase, significant increases were observed in the LPS (*P* = 0.0003), LPS + CAP 25 μM (*P* = 0.0383), and LPS + CAP 50 μM (*P* = 0.0002) groups compared to the control.

**Fig. 4:**
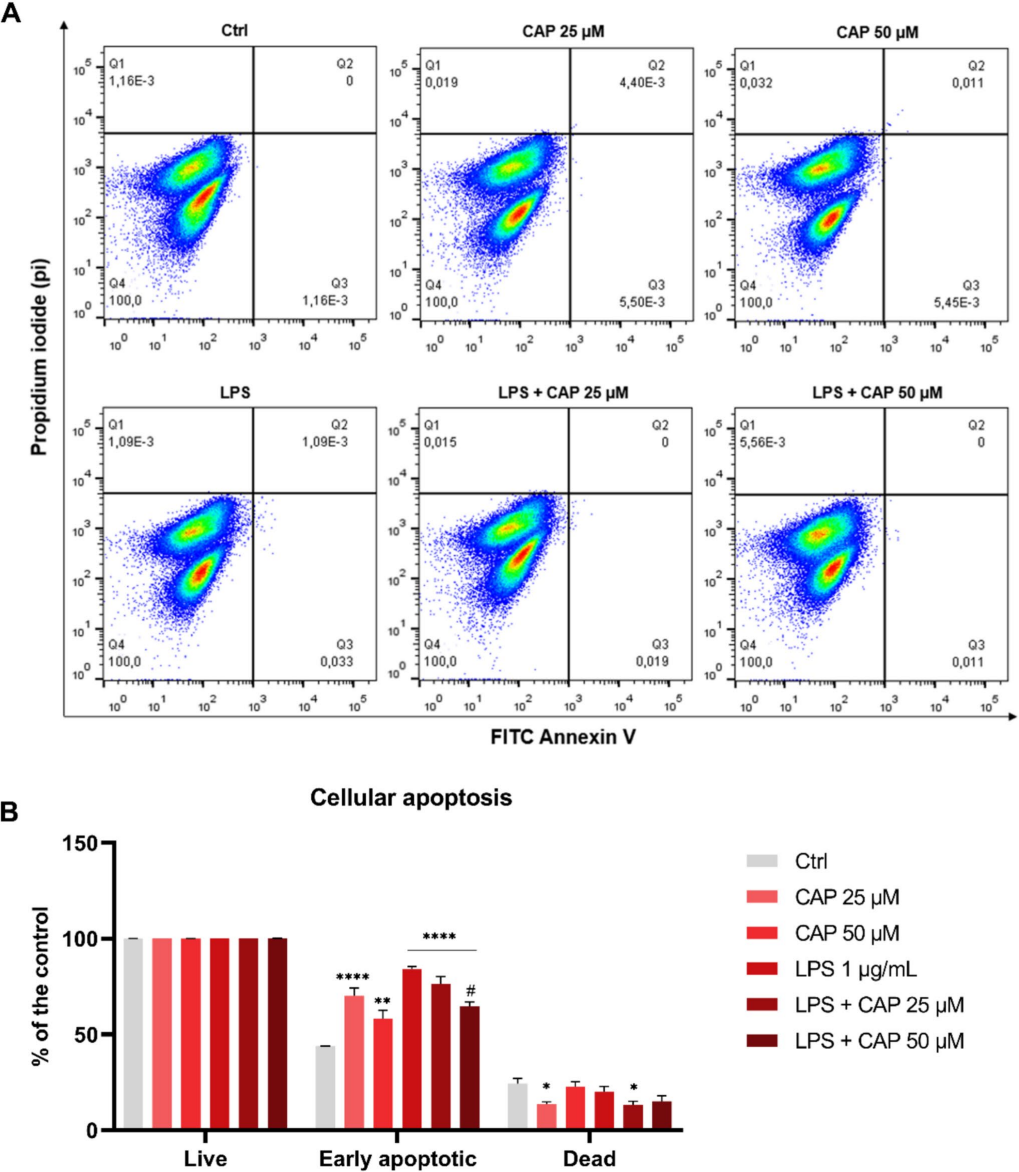
Apoptosis profile of microglial cells (BV-2) treated with or without LPS and capsaicin (25 μM and 50 μM) for 24 hours. Representative images of control and treatment groups (**A**). Percentage of live, early apoptotic and dead cells in relation to the control group (*n* = 3) (**B**). Ctrl = negative control; LPS = lipopolysaccharide; CAP = capsaicin. Values are expressed as mean ± standard error. Statistical significance when *P* < 0.05 (two-way ANOVA followed by Tukey's multiple comparisons test). * Compared to the control group (*P < 0.05, **P < 0.01, and ****P < 0.0001). # Compared to LPS group (#*P* < 0.05)

In the apoptosis analysis (Fig. 4B), the percentage of cells in early apoptosis increased in all experimental groups (CAP 25 μM, *P* < 0.0001; CAP 50 μM, *P* = 0.0012; LPS, *P* < 0.0001; LPS + CAP 25 μM, *P* < 0.0001; LPS + CAP 50 μM, *P* < 0.0001). However, it was reduced in the group treated with LPS + CAP 50 μM (*P* < 0.0001) compared to the LPS group. Regarding the percentage of dead cells, a reduction was observed in the groups treated with CAP 25 μM (*P* = 0.0275) and LPS + CAP 25 μM (*P* = 0.0126) compared to the control.

### Capsaicin attenuates LPS-induced production of oxidative molecules in BV-2 cells

**Fig. 5:**
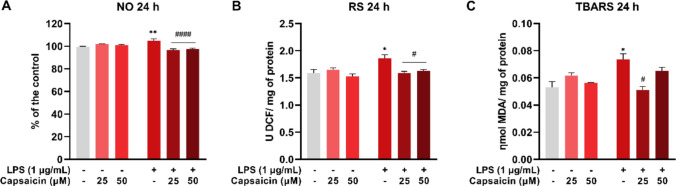
Oxidative parameters in microglial cells (BV-2) treated with or without LPS and capsaicin (25 μM and 50 μM) for 24 hours. Nitric oxide (NO) levels in supernatant (**A**). Generation of reactive species (RS) in cells (**B**). Quantification of thiobarbituric acid reactive substances (TBARS) in cells (**C**). Values are expressed as mean ± standard error. Statistical significance when *P* < 0.05 (two-way ANOVA followed by Tukey's multiple comparisons test). * Compared to the control group (*P < 0.05, ** *P* < 0.01). # Compared to LPS group (#*P* < 0.05 and #### *P* < 0.0001)

The LPS group showed an increase in nitric oxide levels, as indicated by the accumulation of nitrite in the supernatant, compared to the control (*P* = 0.0011). The LPS + CAP 25 μM and LPS + CAP 50 μM groups exhibited a reduction in these levels compared to the LPS group (*P* < 0.0001), returning to levels similar to the control (Fig. 5A). Additionally, an increase in reactive species levels was observed in the LPS group compared to the control (*P* = 0.0261). Capsaicin treatment reduced these values compared to the LPS group (LPS + CAP 25 μM, *P* = 0.0129; LPS + CAP 50 μM, *P* = 0.0310) (Fig. 5B). Furthermore, the LPS group showed an increase in lipid peroxidation (*P* = 0.0154), which was attenuated in the LPS + CAP 25 μM group (*P* = 0.0045) compared to the LPS group (Fig. 5C).

### Capsaicin reduces LPS-induced inflammation in BV-2 cells

**Fig. 6:**
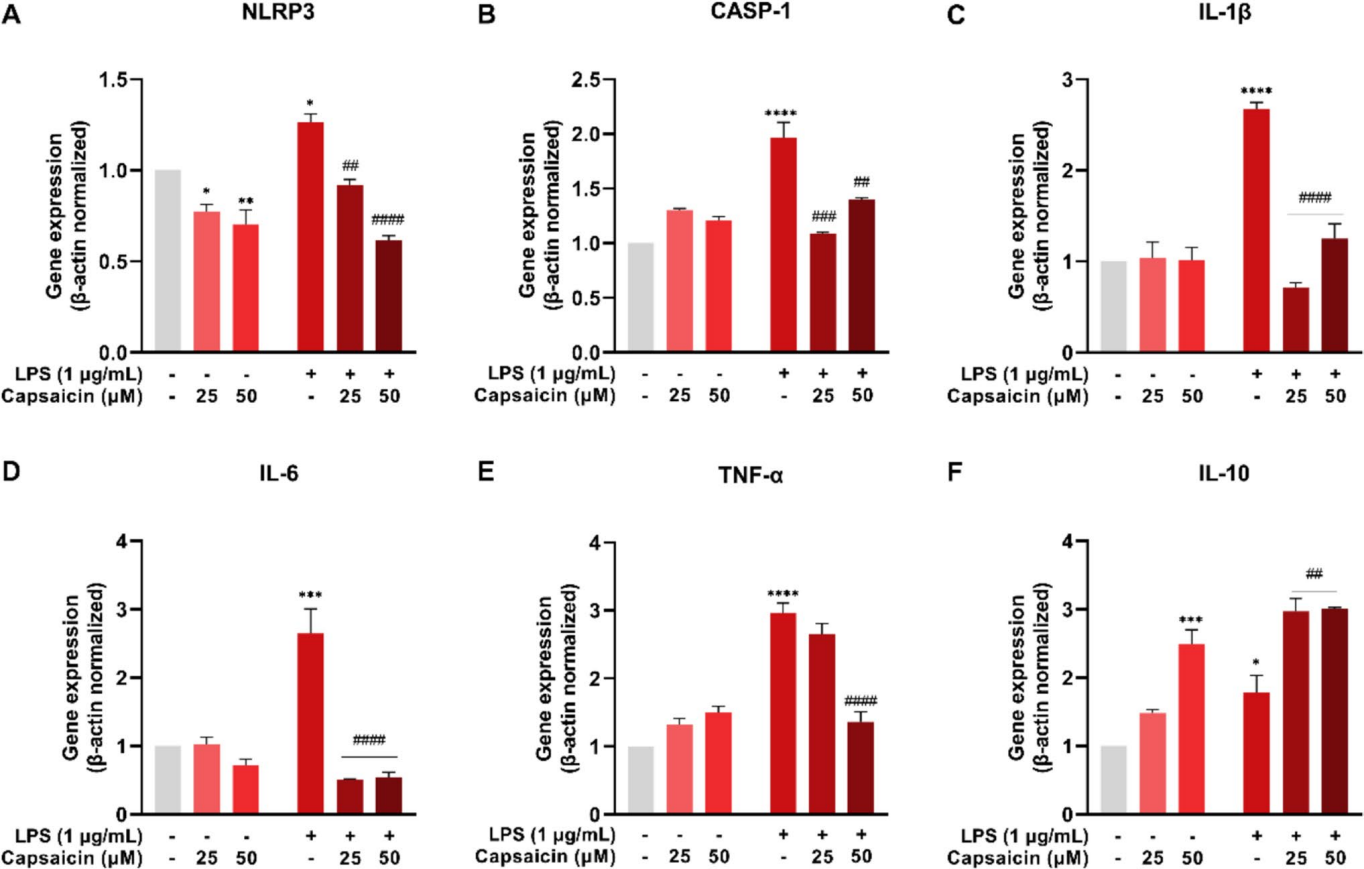
Gene expression profile of microglial cells (BV-2) treated with or without LPS and capsaicin (25 μM and 50 μM) for 24 hours. Gene expression of NLRP3 (**A**), CASP-1 (**B**), IL-1β (**C**), IL-6 (**D**) TNF-α (**E**) and IL-10 (**F**) evaluated by qRT-PCR. Values are expressed as mean ± standard error. Statistical significance when *P* < 0.05 (two-way ANOVA followed by Tukey's multiple comparisons test). * Compared to the control group (*P < 0.05, **P < 0.01, ***P < 0.001, and ****P < 0.0001). # Compared to LPS group (#*P* < 0.05, ##*P* < 0.01, ###*P* < 0.001, and ####*P* < 0.0001)

NLRP3 gene expression was reduced in the CAP 25 μM (*P* = 0.0299) and CAP 50 μM (*P* = 0.0045) groups compared to the control. The LPS group showed an increase in NLRP3 expression (*P* = 0.0112), which was attenuated in the LPS + CAP 25 μM (*P* = 0.0013) and LPS + CAP 50 μM (*P* < 0.0001) groups compared to the LPS group (Fig. 6A). CASP-1 expression increased in the LPS group (*P* < 0.0001) relative to the control, and was reduced in the LPS + CAP 25 μM (*P* = 0.0002) and LPS + CAP 50 μM (*P* = 0.0021) groups compared to the LPS group (Fig. 6B).

Furthermore, an increase in the expression of the pro-inflammatory cytokines IL-1β (*P* < 0.0001), IL-6 (*P* = 0.0001) and TNF-α (*P* < 0.0001) was observed in the LPS group. Capsaicin treatment reduced the expression of IL-1β and IL-6 (LPS + CAP 25 μM, *P* < 0.0001; LPS + CAP 50 μM, *P* < 0.0001) compared to the LPS group (Fig. 6C and D). The expression of TNF-α was reduced only in the LPS + CAP 50 μM group (*P* < 0.0001) (Fig. 6E). IL-10 expression was increased in the CAP 50 μM (*P* = 0.0001) and LPS (*P* = 0.0417) groups compared to the control, as well as an increase in the LPS + CAP 25 μM (*P* = 0.0022) and LPS + CAP 50 μM (*P* = 0.0017) groups (Fig. 6F).

### Capsaicin modulates the activity of purinergic enzymes in LPS-stimulated BV-2 cells

**Fig. 7:**
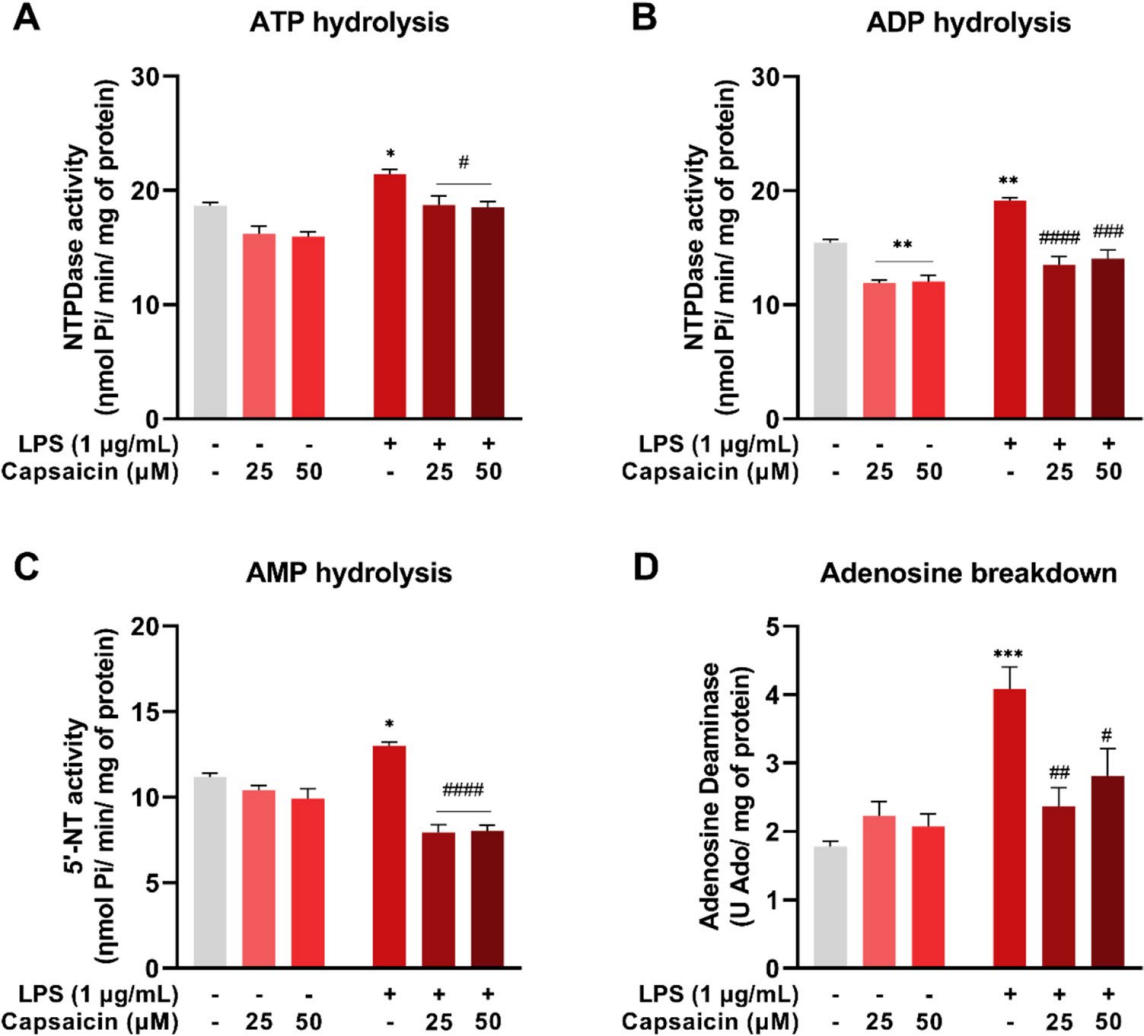
Purinergic ectoenzyme activity in microglial cells (BV-2) treated with or without LPS and capsaicin (25 μM and 50 μM) for 24 hours. NTPDase activity using ATP (**A**) and ADP (**B**) as substrates. 5´-nucleotidase (5’-NT) activity using AMP (**C**) as substrate. Adenosine deaminase (ADA) activity (**D**) using adenosine as substrate. Values are expressed as mean ± standard error. Statistical significance when *P* < 0.05 (two-way ANOVA followed by Tukey's multiple comparisons test). * Compared to the control group (*P < 0.05, **P < 0.01 and ***P < 0.001). # Compared to LPS group (#*P* < 0.05, ##*P* < 0.01, ###*P* < 0.001, and #### *P* < 0.0001)

In ATP hydrolysis, an increase was observed in the LPS group (*P* = 0.0292) compared to the control. Conversely, the LPS + CAP 25 μM (*P* = 0.0340) and LPS + CAP 50 μM (*P* = 0.0225) groups exhibited a decrease in these levels relative to the LPS group (Fig. 7A). Regarding ADP hydrolysis, a reduction was observed in the CAP 25 μM (*P* = 0.0023) and CAP 50 μM (*P* = 0.0030) groups, whereas the LPS group showed a significant increase (*P* = 0.0017) compared to the control. This increase was attenuated in the LPS + CAP 25 μM (*P* < 0.0001) and LPS + CAP 50 μM (*P* = 0.0003) groups (Fig. 7B).

AMP hydrolysis was elevated in the LPS group (*P* = 0.0207), but this effect was reversed in the LPS + CAP 25 μM (*P* < 0.0001) and LPS + CAP 50 μM (*P* < 0.0001) groups (Fig. 7C). Additionally, adenosine deaminase activity increased in the LPS group (*P* = 0.0002) and decreased in the LPS + CAP 25 μM (*P* = 0.0042) and LPS + CAP 50 μM (*P* = 0.0443) groups (Fig. 7D). These results indicate that LPS increases the activity of purinergic enzymes, and this effect is normalized by capsaicin treatment.

### Capsaicin modulates the gene expression of purinergic receptors in LPS-stimulated BV-2 cells

**Fig. 8:**
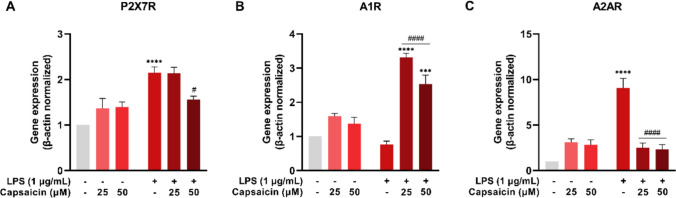
Gene expression profile of purinergic receptors in microglial cells (BV-2) treated with or without LPS and capsaicin (25 μM and 50 μM) for 24 hours. Gene expression of P2X7R (**A**), A1R (**B**) and A2AR (**C**) evaluated by qRT-PCR. Values are expressed as mean ± standard error. Statistical significance when *P* < 0.05 (two-way ANOVA followed by Tukey's multiple comparisons test). * Compared to the control group (***P < 0.001 and ****P < 0.0001). # Compared to LPS group (#*P* < 0.05 and ####*P* < 0.0001)

P2X7R gene expression was increased in the LPS group (*P* < 0.0001) compared to the control. Treatment with LPS + CAP 50 μM (*P* = 0.0251) reduced this expression relative to the LPS group (Fig. 8A). A1R expression was elevated in the LPS + CAP 25 μM (*P* < 0.0001) and LPS + CAP 50 μM (*P* < 0.0001) groups (Fig. 8B). Regarding A2AR, an increase in expression was observed in the LPS group (*P* < 0.0001) compared to the control, and a reduction in expression was observed in the LPS + CAP 25 μM (*P* < 0.0001) and LPS + CAP 50 μM (*P* < 0.0001) groups compared to the LPS group (Fig. 8C).

### Homology modeling and molecular docking

**Fig. 9:**
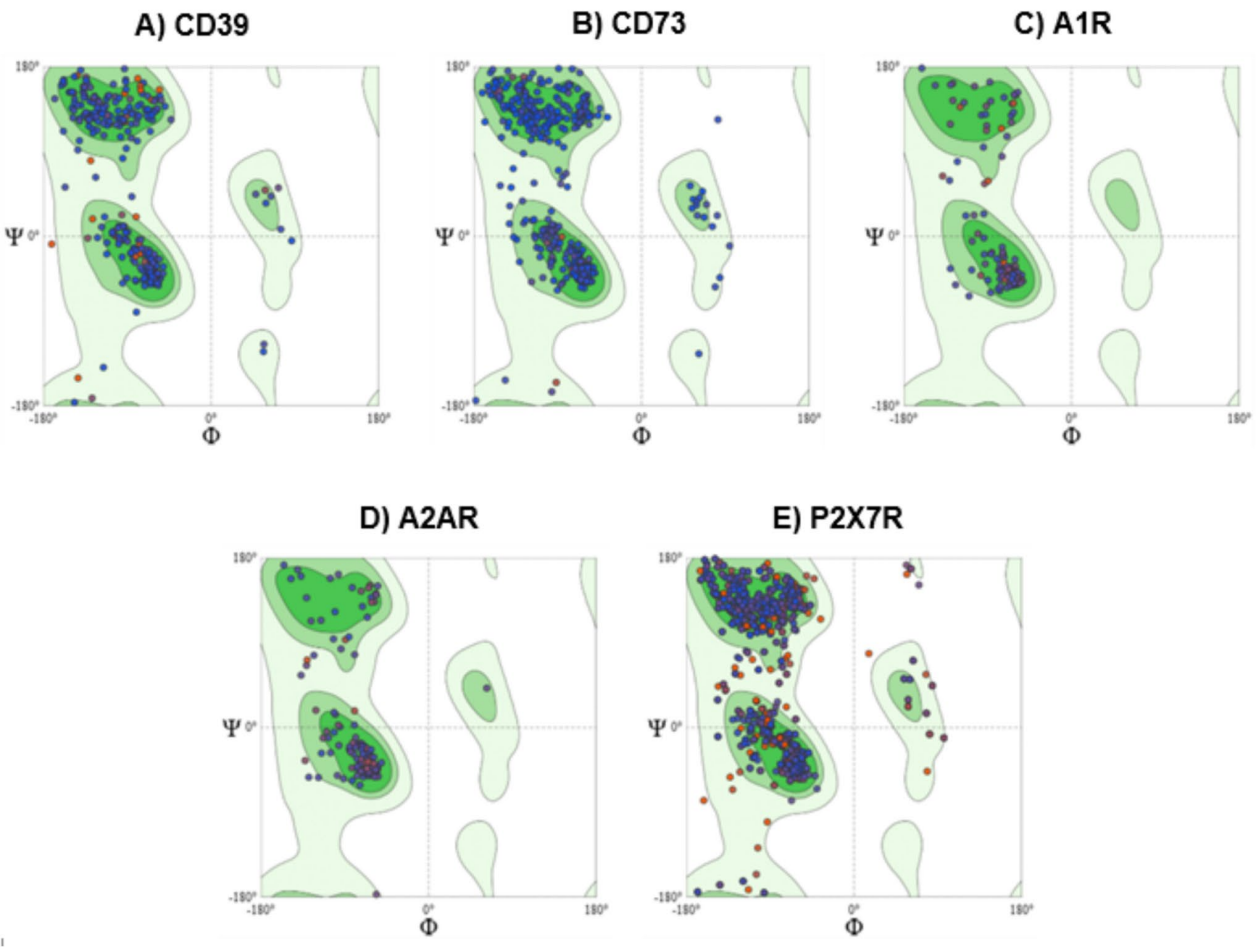
Ramachandran plots of homology models generated by SWISS-MODEL for purinergic targets. The plots display the distribution of backbone dihedral angles (ϕ and ψ) for residues in the modeled structures CD39 (**A**), CD73 (**B**), A1R (**C**), A2AR (**D**) and P2X7R (**E**). The residues are predominantly located within the favored (dark green) and additionally allowed (light green) regions, which indicates the predicted models have good stereochemical quality and structural reliability

All the models showed favorable results for structural validation, both by Ramachandran map and MolProbity Score [42]. The percentages of residues located in favorable regions were 96.38%, 95.78%, 97.70%, 97.67% and 93.42% for CD39, CD73, A1R, A2AR and P2X7R, respectively (Fig. 9). The MolProbity Scores obtained for the same models were 1.56, 0.93, 0.56, 1.21 and 1.19, indicating good structural quality for all of them.

The binding affinity predicted by docking was negative for all enzymes and purinergic receptors (Table 4), indicating spontaneous interactions with capsaicin [43]. However, when comparing these values with the binding affinities of the native substrates obtained by redocking, it was observed that capsaicin showed more expressive affinities for the A2AR and A1R receptors, as well as for the ADA enzyme, with ∆G_bind_ values of −7.7, −7.3 and −7.1 kcal•mol^−1^, respectively. These results suggest a greater competitive potential of capsaicin on these specific targets.

**Table 4:** Free binding energy (theoretical ∆G_bind_ in kcal•mol^−1^) of capsaicin and the native substrates (ATP, ADP, AMP and adenosine) with the purinergic enzymes CD39, CD73 and ADA and with the purinergic receptors A1R, A2AR and P2X7R

Target	∆G_bind_ Capsaicin (kcal•mol^−1^)	Native Substrate	ΔG_bind_ Substrate (kcal•mol^−1^)	Difference (Capsaicin—Substrate)	Competition Potential
CD39	−7.2	ATP/ADP	− 10.1/−9.8	+ 2.9/+ 2.6	Low
CD73	−7.6	AMP	−7.7	0.1	Moderate
ADA	−7.1	Adenosine	−6.4	**−0.7**	**High**
A1R	−7.3	Adenosine	−6.4	**−0.9**	**High**
A2AR	−7.7	Adenosine	−6.4	**−1.3**	**Very high**
P2X7R	−6.2	ATP	−6.7	0.5	Moderate

**Fig. 10:**
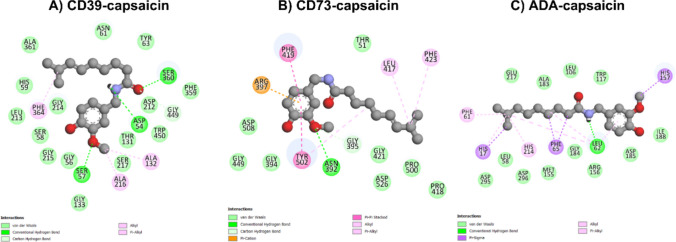
2D interaction diagrams of capsaicin bound to purinergic targets generated using Discovery Studio. The diagrams illustrate the molecular interactions between capsaicin and selected binding site residues for CD39 (**A**), CD73 (**B**), and ADA (**C**). Different interaction types are color-coded: conventional hydrogen bonds (green), carbon hydrogen bonds (light green), alkyl and π-alkyl (light pink), π-sigma interactions (purple), π-cation (orange), and π-π stacked interactions (pink). These interactions highlight the key residues involved in ligand stabilization within the binding pockets of each protein

**Fig. 11:**
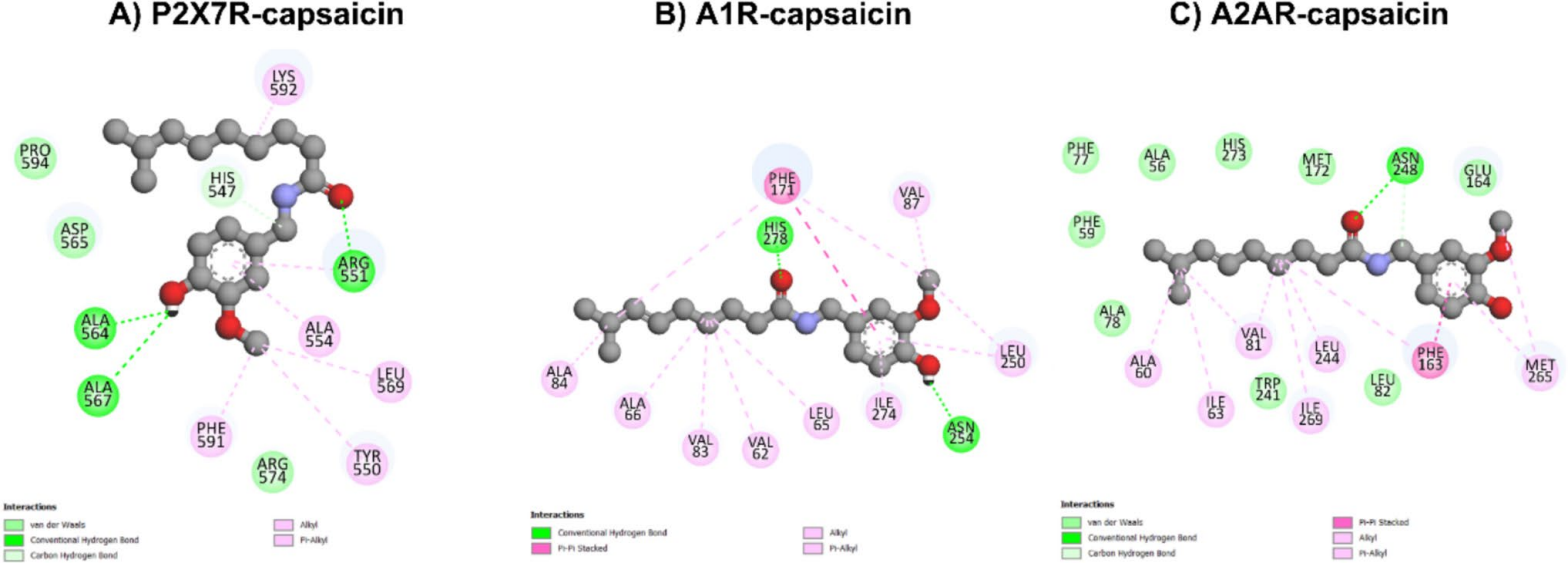
2D interaction diagrams of capsaicin bound to purinergic targets generated using Discovery Studio. The diagrams illustrate the molecular interactions between capsaicin and selected binding site residues for P2X7R (**A**), A1R (**B**), and A2AR (**C**). Different interaction types are color-coded: conventional hydrogen bonds (green), carbon hydrogen bonds (light green), alkyl and π-alkyl (light pink), and π-π stacked interactions (pink). These interactions highlight the key residues involved in ligand stabilization within the binding pockets of each protein

The molecular interactions observed between capsaicin and purinergic targets revealed a variety of contacts, indicating significant binding potential (Figs. 10 and 11). Conventional hydrogen bonds with specific residues were observed in all targets, including Ser360, Ser57 and Asp54 (CD39); Asn392 (CD73); Leu62 (ADA); His278 and Asn254 (A1R); Asn248 (A2AR); and Arg551, Ala564 and Ala567 (P2X7R). This reinforces the specificity of the interaction and reflects the stability of the complexes, as demonstrated by the negative ∆G, since hydrogen bonds are considered strong bonds [44]. Additionally, hydrogen bonds mediated by carbon atoms were identified in the CD39, CD73, A2AR and P2X7R targets, interacting with the residues Gly449, Gly395, Asn248 and His547. The π-cation interaction was exclusive to CD73 and involved the Arg397 residue. Meanwhile, π-sigma interactions were only observed in the ADA enzyme with the aromatic residues His17, Phe65 and His157.

Hydrophobic alkyl interactions were widely distributed among the targets, particularly between Ala216 and Ala132 (CD39), Leu417 (CD73), Leu62 (ADA), Lys592 and Leu569 (P2X7R), and multiple residues in the A1R and A2AR receptors (e.g., Ile63, Val87, and Met265). The π-alkyl interactions involving aromatic residues or the aromatic structure of capsaicin occurred with residues such as Phe364 (CD39), Tyr502 (CD73), Phe61 and His17 (ADA), Phe171 and Leu250 (A1R), Met265 and Phe163 (A2AR), and Tyr550 and Phe591 (P2X7R). Aromatic stacking (π-π stacked) was identified in CD73, ADA, and A2AR with residues such as Tyr502, Phe171, and Phe163. These findings suggest that aromatic interactions significantly contribute to stabilizing the complexes. Finally, van der Waals interactions were observed in all targets, involving a variety of residues, which indicates that non-specific forces play an important role in capsaicin recognition by different purinergic targets.

## Discussion

Neuroinflammation is a critical process in various pathologies affecting the CNS and is associated with microglial activation and the modulation of signaling pathways, such as the purinergic system [2, 45, 46]. Capsaicin is notable for its ability to modulate inflammatory pathways [19, 47]. The findings of this study indicate that capsaicin exerts an immunomodulatory effect on BV-2 microglial cells. Additionally, it modulates the activity of enzymes and purinergic receptors, suggesting its potential to influence extracellular signaling processes mediated by nucleotides and nucleosides.

The concentration–response curve of capsaicin was evaluated to determine safe and physiologically relevant concentrations, avoiding potential pro-inflammatory or cytotoxic effects induced by capsaicin alone. The results showed that capsaicin did not compromise cell viability at concentrations up to 50 μM, and no significant changes in nitric oxide production were observed. Other studies in microglia have reported no cytotoxicity within this concentration range [3, 48], and investigations in neuronal cells have shown similar findings [23, 49]. Therefore, we selected concentrations of 25 and 50 μM, as capsaicin has been demonstrated to effectively modulate inflammatory responses within this range without inducing cytotoxicity.

LPS-induced microglial activation is a widely used model for studying neuroinflammation, as it activates pro-inflammatory pathways via the Toll-Like Receptor 4 (TLR4) [50, 51]. The concentration of LPS (1 μg/mL) used in this study did not significantly affect cell viability, consistent with previously reported data [52]. In the apoptosis analysis, an increase in the percentage of cells in early apoptosis was observed in all experimental groups compared to the control. However, the majority of cells remained viable across all groups, indicating a minimal impact on overall cell survival.

LPS induced alterations in the cell cycle of BV-2 cells, as evidenced by a decrease in the G0-G1 phase and an increase in the S and G2-M phases. These findings suggest proliferative stimulation associated with inflammatory activation [53]. Capsaicin treatment modulated these changes, particularly by reducing the percentage of cells in the S phase, indicating a potential antiproliferative effect. The antiproliferative action of capsaicin has been demonstrated in studies with cancer cells, where its effect increases proportionally with concentration. However, this activity shows considerable variability among different cell lines [54–56].

Gene expression analysis confirmed the activation of inflammatory pathways, including inflammasome components such as NLRP3 and caspase-1, pro-inflammatory cytokines IL-1β, IL-6, and TNF-α, and oxidative markers NO, ER, and MDA. LPS stimulation increased these markers, while capsaicin treatment significantly reduced them, highlighting its anti-inflammatory and antioxidant properties. Consistent findings in macrophages and BV-2 cells also demonstrate that capsaicin inhibits LPS-induced secretion of IL-1β, IL-6, TNF-α, and NO [3, 57].

The NLRP3 inflammasome is a multiprotein complex of the innate immune system that is formed in response to pathogenic stimuli or signals of cellular damage. Its activation occurs in two stages. The first stage, known as priming, involves the recognition of ligands such as LPS, which activate the NF-κB pathway and promote the transcription of NLRP3 and the precursor form of IL-1β [58, 59]. The second stage, activation, is triggered by intracellular signals such as K^+^ efflux, reactive species release, mitochondrial dysfunction, lysosomal damage, or ATP release [58, 60]. These signals induce the inflammasome formation by promoting NLRP3 activation and the recruitment of the adaptor protein ASC and caspase-1. Once activated, caspase-1 cleaves IL-1β into its mature form, amplifying the inflammatory response [59].

Capsaicin has been shown to modulate inflammation by inhibiting NF-κB translocation to the nucleus [3, 61]. NF-κB regulates the expression of pro-inflammatory genes such as TNF-α, IL-1β, IL-6, NLRP3, inducible nitric oxide synthase (iNOS), and cyclooxygenase-2 (COX-2). By blocking NF-κB signaling, capsaicin prevents the activation of these genes [3, 61–63]. Capsaicin also upregulates IL-10 expression, an anti-inflammatory cytokine that suppresses pro-inflammatory mediator production and promotes an anti-inflammatory immune response [64–66]. These findings suggest that capsaicin not only inhibits inflammation but also fosters a return to homeostasis and neuroprotection during inflammatory processes [19, 21, 67].

The NF-κB pathway is activated by signaling through TLR receptors, which also promote the release of ATP into the extracellular environment. Extracellular ATP activates P2X7R, providing the second signal required for inflammasome activation [13, 68]. In addition to inhibiting the NLRP3/CASP-1/IL-1β pathway, our results show that capsaicin reduces P2X7R gene expression at the highest concentration tested.

Molecular docking results indicate that capsaicin exhibits moderate affinity for P2X7R (−6.2 kcal•mol^−1^), a value close to that of the endogenous ligand ATP (−6.7 kcal•mol^−1^). These similar values suggest that capsaicin may compete with ATP for the binding site or interact allosterically with the receptor, thereby modulating its conformation. This interaction could directly affect the dynamics of ion channel opening and influence Ca^2+^ and sodium (Na^+^) influx, as well as the release of inflammatory mediators associated with P2X7R activation. From a pharmacological perspective, these findings suggest that capsaicin could modulate P2X7R-mediated inflammatory processes. It can also be hypothesized that this modulation is related to capsaicin's antioxidant and anti-inflammatory properties, which contribute to the attenuation of cellular stress and, consequently, reduce ATP release into the extracellular environment [20, 69, 70].

In addition to interacting with purinergic receptors, the extracellular ATP released in response to inflammatory stimuli is hydrolyzed by purinergic ectoenzymes into adenosine, a nucleoside with anti-inflammatory effects [16, 18]. The activity of NTPDase, 5'-NT, and ADA were increased in the LPS group, indicating elevated levels of extracellular nucleotides and nucleosides. Studies in macrophages and monocytes have shown that LPS induces the immediate release of ATP through the pannexin-1 channel [71]. In the groups exposed to LPS and treated with capsaicin, enzyme activity was reduced, approaching baseline levels observed in the control group. These findings suggest that capsaicin attenuates ATP release by mitigating inflammation.

The immunomodulatory effects of adenosine are primarily mediated through its activation of P1-type receptors.

Among these subtypes, A1R and A2AR play pivotal roles in regulating microglial activity and are both activated by physiological levels of adenosine [70, 72]. However, these receptors exert divergent effects: A1R is associated with anti-inflammatory effects, whereas A2AR can play a pro-inflammatory role in certain contexts [16]. The LPS group exhibited a marked increase in A2AR expression. In contrast, capsaicin treatment decreased A2AR expression and increased A1R expression compared to the LPS group. This regulatory profile suggests the promotion of anti-inflammatory adenosinergic signaling.

In microglia, A1R has been shown to actively suppress inflammatory activation. This effect is mediated through intracellular signaling pathways, including negative modulation of inflammatory transcription factors such as NF-κB, resulting in reduced expression of pro-inflammatory cytokines and other neurotoxic mediators [17]. In addition, A1R activation supports the maintenance of synaptic integrity and neuronal function. Under physiological and pathological conditions, A1R promotes the release of trophic factors, such as nerve growth factor (NGF), highlighting its central role in protecting neural tissue during inflammatory processes [73, 74].

A2AR is prominently expressed in striatal neurons and minimally in glial cells and neurons of other brain regions under normal conditions [74]. During brain injury or inflammation, A2AR expression significantly increases in microglial cells [74, 75], facilitating the release of pro-inflammatory cytokines and promoting an amoeboid morphology associated with microglial activation and an amplified immune response [75]. A2AR enhances the synthesis of inflammatory molecules by modulating glutamate release, NMDA receptor activity, and glial reactivity [76, 77]. Studies have shown that A2AR antagonists can suppress microglial activation, highlighting its pro-inflammatory role in pathological conditions [74, 76, 78, 79].

Molecular docking analyses indicate that capsaicin exhibits high binding affinity for ADA (−7.1 kcal•mol^−1^), A1R (−7.3 kcal•mol^−1^), and A2AR (−7.7 kcal•mol^−1^). These results suggest that the compound has a high potential to compete with the endogenous ligand, adenosine (−6.4 kcal•mol^−1^). This could directly impact the modulation of purinergic signaling by interfering with adenosine binding to its receptors or its degradation by ADA. Among these targets, A2AR showed the most favorable interaction with capsaicin, indicating a strong binding affinity. This ability of capsaicin to modulate purinergic signaling may have relevant implications for neuroinflammatory processes. By interacting with adenosine P1 receptors, capsaicin may influence microglial activation and cytokine release.

Capsaicin is a well-known agonist of TRPV1, a non-selective cation channel expressed in microglia that contributes to the regulation of neuroinflammation [80]. Although TRPV1 activation may mediate some of capsaicin’s effects, studies indicate that its anti-inflammatory actions in microglia can also occur through TRPV1-independent mechanisms [63, 81–83]. In this study, we focused on its overall anti-inflammatory actions in microglia, without directly assessing TRPV1 involvement. However, the effects observed in the present study may still involve TRPV1, and further investigations are needed to clarify its specific contribution.

From a pharmacokinetic perspective, capsaicin exhibits widespread distribution throughout the body due to its high lipophilicity. However, it undergoes first-pass metabolism and hepatic biotransformation, which reduce its oral bioavailability [47, 84]. Nonetheless, studies in rodents have shown that capsaicin can cross the blood–brain barrier, reaching the CNS at detectable concentrations and exerting neuroprotective effects in various contexts [22–24, 49, 84–86]. These findings suggest that, despite its pharmacokinetic limitations, capsaicin has translational potential for modulating neuroinflammatory processes in vivo, supporting the relevance of the results obtained in the in vitro model used in this study.

## Conclusion

The present findings indicate that capsaicin effectively modulates neuroinflammation in microglial cells by inhibiting the NLRP3/CASP-1/IL-1β pathway and regulating the purinergic system. Capsaicin has been shown to downregulate P2X7R and A2AR expression while upregulating A1R expression, reinforcing its potential to create a neuroprotective environment. Additionally, molecular docking analyses indicate that capsaicin can directly interact with adenosine receptors, suggesting its potential to modulate purinergic signaling through multiple mechanisms, including receptor binding and transcriptional regulation. Collectively, these results position capsaicin as a promising agent for controlling microglial activation and purinergic signaling, with potential therapeutic relevance for neuroinflammatory conditions. However, further research is needed to elucidate the molecular mechanisms underlying capsaicin’s modulation of the purinergic system.

## Data Availability

No datasets were generated or analysed during the current study.
